# Bioelectronic medicine: updates, challenges and paths forward

**DOI:** 10.1186/s42234-019-0018-y

**Published:** 2019-01-22

**Authors:** Valentin A. Pavlov, Kevin J. Tracey

**Affiliations:** 1 Center for Biomedical Science and Center for Bioelectronic Medicine, The Feinstein Institute for Medical Research, Northwell Health System, Manhasset, NY USA; 2 Donald and Barbara Zucker School of Medicine at Hofstra/Northwell, Hempstead, NY USA

It is an exciting time for the expanding field of Bioelectronic Medicine*. O*ngoing innovative preclinical research and development provide a rationale for studying new diagnostic and treatment approaches in inflammatory and autoimmune diseases, diabetes, paralysis and other disorders. *Bioelectronic Medicine,* on the Springer/Nature BMC platform reflects the commitment to a necessary forum for publishing cutting edge primary research, timely reviews and other relevant publications. Here we comment on the recent content of *Bioelectronic Medicine* (published in 2018) that captured many important aspects of the current state of the field. Our aims are to trigger multidisciplinary discussions and to inspire future research efforts that will contribute to advancing the field.

Bioelectronic medicine continues to benefit from improved technology for neural modulation generated by the multidisciplinary team work of material scientists, electrical engineers, mechanical engineers, neuroscientists and colleagues from other disciplines (Cogan, 2008; Datta-Chaudhuri et al., 2016; Negi et al., 2010; Olafsdottir et al., 2018). The latest advances in flexible electrodes and other components for neuromodulation of peripheral nerves were outlined in *Bioelectronic Medicine* by Giagka and Serdijn (Giagka & Serdijn, 2018). They describe the current technological state of these “devices that use electricity to regulate biological processes, treat diseases, or restore lost functionality” (Giagka & Serdijn, 2018). They point to several challenges that remain, including optimizing hybrid configurations of electrodes and devices, and miniaturization. Substantial efforts have focused on polymer processing as a core procedure in developing flexible implantable devices with high biocompatibility. The authors note that electrode materials have shifted from “noble metals to advanced coatings, constructed by either different materials, or different geometries.” Testing these new modules in vivo has generated promising results related to both recording and stimulating peripheral nerve activity. The path forward will reduce impedances, increase electrochemically active surfaces, optimize electrode integration, improve specificity and further miniaturize this technology for chronic neuromodulation (Giagka & Serdijn, 2018). In a related review Bettinger (Bettinger, 2018) focuses on the need to optimize flexible electronics for peripheral nerve interfaces to respond to the increased demand for reliability along with improved performance for chronically implanted bioelectronic devices. The author points to the need for novel materials to produce flexible and stretchable substrates yielding optimal signal transduction across the tissue-electrode interface, new microfabrication techniques, and improved device packaging. Better understanding of the foreign body response mechanisms to implanted devices will also be key in informing reliable tissue-device integration. An important forum bringing together neuroengineers and professionals from related disciplines is the biennial Cleveland Neural Engineering Workshop (NEW). A short report by Anderson et al., summarizes the main outcomes from a previous meeting (Anderson et al., 2018). Neural engineering and other disciplines are collaborating on more efficient communications with users, regulatory guidance, network building (clinical practice), industrial feedback, resources, engagement, and advocacy (Anderson et al., 2018).

The vagus nerve continues to hold a specific and important place in bioelectronic medicine. Pioneering research on the role of the vagus nerve in the regulation of immune responses and inflammation that started more than 20 years ago led to several important developments in bioelectronic medicine (Borovikova et al., 2000; Tracey, 2002; Pavlov et al., 2018). One is the successful use of vagus nerve stimulation (VNS) in the treatment of human inflammatory and autoimmune diseases (Koopman et al., 2016; Meregnani et al., 2011; Pavlov & Tracey, 2017). The current experience with using implanted device-generated VNS in a clinical trial with patients with inflammatory bowel disease (Crohn’s disease) was summarized by Bonaz (Bonaz, 2018). The author outlines important findings in the context of our improved understanding of the functional anatomy of the vagus nerve, new clinical knowledge of VNS for epilepsy and depression and remaining challenges (Bonaz, 2018). These include finding optimal regimens (frequency, pulse width, waive forms, and duration) of chronic VNS, further miniaturization of the devices and targeting specificity. He also summarizes the pros and cons of invasive (through implanted devices) and non-invasive VNS, including transcutaneous VNS of the auricular branch of the vagus nerve, or the use of devices for cervical vagus nerve stimulation (Lerman et al., 2016) as therapeutic approaches (Bonaz, 2018). Preclinical and clinical research going hand in hand is critical for advancing the field of Bioelectronic Medicine. Studies on the immunomodulatory role of the vagus nerve are an excellent example of this symbiotic relationship.

An exciting new area of vagus nerve research addresses the need to reliably measure and interpret neural signaling. Silverman et al. reported a standardized method for determining vagus nerve activity in mice by recording compound action potentials using a cuff electrode (Silverman et al., 2018). The utility of this method is demonstrated by recording cervical vagus neural activity in response to anesthesia, feeding, or exposure to bacterial endotoxin (Silverman et al., 2018). This approach and other recent advances (Zanos et al., 2018) provide a substantial new insight into the specific electrical activity of the vagus nerve (“neurograms”) in response to cytokines and other characteristic challenges with potential future clinical implications. Another key issue that needs to be addressed is to determine specificity of vagus nerve signaling in terms of fiber activation in response to VNS. The vagus nerve regulates cardiac function, and VNS has been studied as a therapeutic strategy for chronic heart failure (De Ferrari et al., 2011). Qing et al. reported that cardiac activity in rats is well correlated with activation of a specific neuronal population, classified as B fibers and characterized as vagus efferents (Qing et al., 2018). These findings may be of interest for further systematic investigations of the effects of VNS and its specific physiological effects (Qing et al., 2018). In addition to the vagus nerve, interesting research focusing on clinically-relevant physiological regulation of neural activity of other peripheral nerves was also published (Jiman et al., 2018). Previous findings have indicated that renal denervation has beneficial effects in treating drug-resistant hypertension (Pan et al., 2015). There is also a growing interest in the role of the renal nerves in the regulation of glucose homeostasis (Pan et al., 2015). Using a cuff electrode positioned to modulate renal nerves residing adjacent to the left renal artery, Jiman et al. (Jiman et al., 2018) reported that stimulation at kilohertz frequencies (1–50 kHz), designed to block action potential propagation, or low frequencies (2–5 Hz), with intravenous administration of a glucose, increased, or respectively lowered, urine glucose excretion (Jiman et al., 2018). This is an interesting avenue for organ-targeted neuromodulation studies aimed at altering glucose excretion for therapeutic benefit. Sanjuan-Alberte et al. provided an overview of current studies on faradaic currents (Sanjuan-Alberte et al., 2018). Faradaic currents are an important, but somewhat neglected form of cellular electrical communication, essential for maintaining homeostasis (Sanjuan-Alberte et al., 2018). The authors point to the possibilities of performing future studies on mechanisms underlying this form of cellular communication with relevance to Bioelectronic Medicine in cancer and other conditions (Sanjuan-Alberte et al., 2018).

Studies providing mechanistic insight into the CNS in health and disease are extremely important for Bioelectronic Medicine (Pavlov et al., 2018; Bouton et al., 2016; Cabrera et al., 2014; George & Aston-Jones, 2010). Brain function modulation has been exploited in Parkinson disease, Alzheimer’s disease, dystonia and other neurodegenerative diseases (Cabrera et al., 2014; George & Aston-Jones, 2010). Approaches, including deep brain stimulation, transcranial magnetic stimulation and other invasive and non-invasive means of neuromodulation are in clinical or exploratory use for treating these disorders (Cabrera et al., 2014; George & Aston-Jones, 2010). Advances in the mechanisms of the neural circuitry involved and network functions will considerably contribute to improving the therapeutic efficacy of these approaches and minimizing potential adverse effects. Ongoing research focuses on better understanding of cortical responses implicated in beneficial effects of subcortical deep brain stimulation. Kibleur and David reviewed current aspects and challenges in using electroencephalography in delineating cortical responses in patients subjected to deep brain stimulation (Kibleur & David, 2018). They also point to the applicability of this approach in advancing our understanding of the functional neuroanatomy of the human brain (Kibleur & David, 2018). The authors also outline some challenges, including the complex post-processing of electroencephalography data and noise correction, and the need for optimizing future efforts by utilizing shared methods in open-source processing toolboxes, and data sharing between international centers. In a related publication Guduru et al. highlighted the potential use of magnetoelectric effect of multiferroic nanoparticles (magnetoelectric nanoparticles) coupled with the ultra-fast and highly sensitive magnetic particle imaging for mapping electric field activity of the brain at the sub-neuronal level and monitoring brain electrical activity in real time (Guduru et al., 2018). Further development and validation of this approach has implications in studying pathogenesis and progression of neurodegenerative diseases and in developing prevention and treatment strategies (Guduru et al., 2018). Paralysis is a devastating condition and current treatment options are very limited (Bouton et al., 2016)**.** Recently, volitional muscle activation in a paralyzed human was successfully achieved using intracortically recorded signals (Bouton et al., 2016). This and other important developments indicated that advanced bioelectronic neuroprosthetic technology and stimulation paradigms have the potential to alleviate the effects of paralyses (Bouton et al., 2016; Angeli et al., 2018; Capogrosso et al., 2016). In a related study (published in *Bioelectronic Medicine)* Zhang et al. provided insight into the utility of wavelet decomposition as a method to extract neural features obtained from a microelectrode array implanted in the motor cortex of a human with tetraplegia (Zhang et al., 2018). The authors demonstrate that neural information from the brain can be reliably recorded for over 3 years after implanting microelectrodes and indicate the use of this method as an important alternative to other currently used neural feature extraction methods (Zhang et al., 2018). These results may further guide the use of brain computer Interfaces with the goal of advanced understanding of the long-term behavior of intracortically-recorded signals. They will also be of interest for optimizing chronic clinical applications involving cortically controlled neuromuscular stimulation to enable multiple hand movements in the same patient (Zhang et al., 2018). Studies on brain function in response to altered oxygen supplies are relevant to many pathophysiological conditions within the scope of Bioelectronic Medicine. As noted by Bailey, the ability to “sense” oxygen and maintain homeostasis is a major driving force in the evolution of the human brain (Bailey, 2018). This author provides an overview of the multiple aspects of the role of oxygen in human evolution and brain development (Bailey, 2018). He summarizes existing knowledge and points to the need for a better understanding of brain mechanisms involved in sensing hypoxia and elaborating redox-signaling defense mechanisms (Bailey, 2018).

Obesity and diabetes are pandemic and present significant challenges to modern society (Mokdad et al., 2003; van Dieren et al., 2010). Better understanding of the role of the nervous system in obesity and diabetes pathophysiology will generate important insights for using neuromodulation and bioelectronic technology in the treatment of these disorders (Pavlov & Tracey, 2012; Pelot & Grill, 2018). Güemes and Georgiou provided an overview of the role of brain and peripheral neural mechanisms in the regulation of glucose homeostasis (Güemes & Georgiou, 2018). They discus on the relevance of this knowledge to developing neuromodulatory approaches to control glucose levels (Güemes & Georgiou, 2018). In a related publication Kovatchev outlined challenges in maintaining strict glycemic control without increasing the risk of hypoglycemia as a central problem in the clinical management of diabetes (Kovatchev, 2018). The author summarizes the substantial progress in advancing diabetes technology. These include improved continuous subcutaneous insulin infusion, developing mathematical models and computer simulation of human metabolic processes, real-time continuous glucose monitoring (CGM), and control algorithms resulting in closed-loop systems known as the “artificial pancreas” (Kovatchev, 2018). He also reviews ongoing clinical trials testing this artificial pancreas technology as wearable home-use devices and notes the FDA approval of the first hybrid closed-loop system (Kovatchev, 2018).

It was estimated that in 2015, 36 million people were blind, and 217 million had moderate or severe vision impairment (Bourne et al., 2017). *Visual prostheses* are medical devices designed to provide some degree of vision to individuals with blindness as a result of severe degeneration or damage to the retina, the optic nerve, or brain damage (Fernandez, 2018). Fernandez summarized the current state of studying electrical stimulation in this context and developing, and using visual prostheses (Fernandez, 2018). The author also outlines challenges in optimizing visual prostheses related to biocompatibility, biotolerability and viability of electrodes and devices in current use. Of note, similar issues have been described in developing technology for peripheral nerve modulation (Giagka & Serdijn, 2018; Bettinger, 2018). Other important questions are related to better understanding brain plasticity associated with the use of visual prostheses (Merabet et al., 2005; Fernandez, 2005) and developing personalized rehabilitative strategies (Fernandez, 2018). The use of optogenetics may offer ways to overcome limitations of currently used electrical stimulation approaches (Fernandez, 2018; Sahel & Roska, 2013).

A growing knowledge of mechanisms of neural regulation in disease pathogenesis will be instrumental for developing new bioelectronic technologies. *Bioelectronic Medicine* will be the platform for innovative research and conceptual developments reflecting the progress in the field.

## Abbreviations

NEW: Neural Engineering Workshop
VNS: Vagus nerve stimulation
